# Anti-Influenza Virus Activity and Phenolic Content of Pomegranate (Punica granatum L.) Peel Extract and Fractions

**Published:** 2019

**Authors:** Mohammad-Taghi Moradi, Ali Karimi, Mehrdad Shahrani, Leila Hashemi, Mohammad-Saleh Ghaffari-Goosheh

**Affiliations:** 1. Medical Plants Research Center, Basic Health Sciences Institute, Shahrekord University of Medical Sciences, Shahrekord, Iran; 2. Cellular and Molecular Research Center, Basic Health Sciences Institute, Shahrekord University of Medical Sciences, Shahrekord, Iran; 3. Clinical Biochemistry Research Center, Basic Health Sciences Institute, Shahrekord University of Medical Sciences, Shahrekord, Iran

**Keywords:** Antiviral Agents, Pomegranate, Punica granatum L

## Abstract

**Background:**

Influenza virus, associated with high level of morbidity and mortality, has been recently considered a public health concern while the choices for the control and treatment of the disease are limited. The present study was conducted to evaluate activity of pomegranate peel extract and its fractions against *Influenza A* virus *in vitro* .

**Methods:**

In this research, ethyl alcohol extract of pomegranate peel was prepared and subjected to fractionation with different polarities. The potential *in vitro* anti-influenza A virus activity of the extract and fractions was assessed using Cytopathic Effect (CPE) reduction assay, Hemagglutinin Assay (HA), and 50% Tissue Culture Infectious Doses (TCID_50_) method in Madin-Darby Canine Kidney (MDCK) cells.

**Results:**

The crude pomegranate peel extract and its n-butanol and ethyl acetate fractions had the highest inhibitory effect against influenza A virus with IC_50_ value of 6.45, 6.07 and 5.6 *μg/ml* in MDCK cells, respectively. Our results also showed that, the production of virus was significantly reduced upon treatment with crude extract, n-butanol and ethyl acetate fractions in a dose-dependent manner (p<0.05).

**Conclusion:**

Based on our results, the ethyl alcohol extract and its polar fractions of pomegranate peel can inhibit influenza A virus replication *in vitro*. Therefore, further characterization of its active ingredients and the mechanism of action should be carried out.

## Introduction

Pomegranate (Punica granatum L., belonging to the Punicaceae family) is one of the oldest edible fruits and is widely cultivated in many tropical and subtropical countries 1, including Iran, Egypt, Russia, Spain, France, China, Japan, Argentina, USA, and India. Pomegranate has been used extensively in the folk medicine of Iranians and many other countries. Many studies have indicated the antioxidant, antiatherogenic, anticancer, anti-inflammatory, antimicrobial and anti-infective effects of pomegranate peel and fruit extracts 2–6.

Although pomegranate peel is sometimes considered an agro-waste, it is indeed as a source of different flavonoids with antibacterial, antiviral, antioxidant, anti-inflammatory and antineoplastic bioactivities 7,8.

One of the most common human respiratory tract pathogens, associated with high morbidity and mortality, namely, influenza virus, has been recently considered a public health concern. Although vaccination is a suitable approach to prevent influenza, this method should be updated to be effective on new subtypes due to constant changes in virus surface 9. Currently, there are two groups of anti-influenza agents available for the management of influenza infection. One class includes amantadine and rimantadine which are matrix protein (M2) ion-channel inhibitors and interfere with viral un-coating within the host cells. They are effective only against influenza virus A with the risk of widespread drug resistance. The other group includes oseltamivir and zanamivir which are Neuraminidase (NA) inhibitors and are widely used in the treatment of both seasonal and pandemic influenza virus infections 10. However, oseltamivir resistant H1N1 strains were found to be circulated since 2007-08 11,12. As the options for the control and treatment of the disease are limited, use of herbal extracts such as pomegranate seems to be an alternative. This research was conducted to evaluate the anti-influenza A virus activity of crude hydro alcoholic extract and the four corresponding fractions of Punica granatum L. peel *in vitro*.

## Materials and Methods

### Plant collection, extraction and fractionation

**Figure 1.**
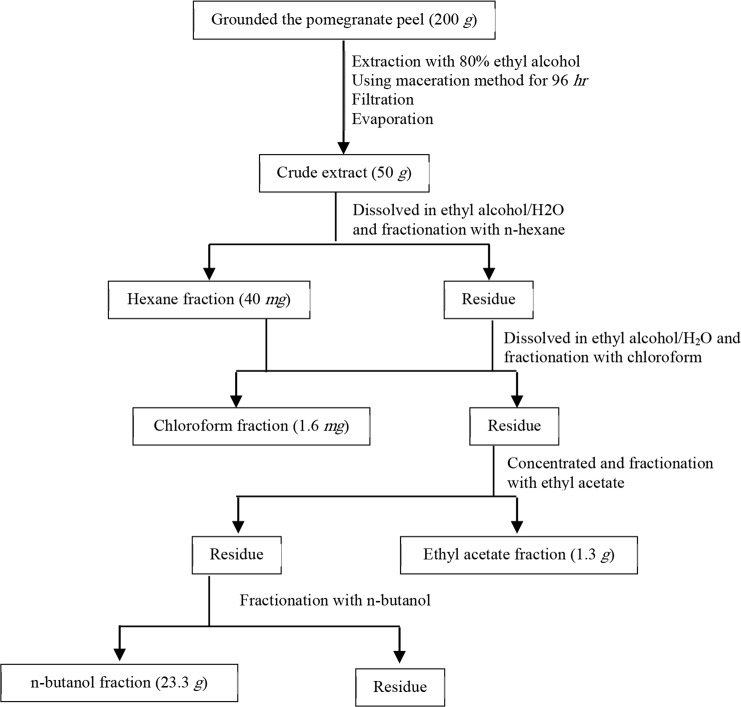
Flow chart for the extraction and fractionation of pomegranate peel

The pomegranate (Punica granatum L.) was from the Malas variant obtained from Shahreza, a central region of Iran (October 2015). Then, in the Herbarium of Medical Plants Research Center of the Shahrekord University of Medical Sciences (Iran), genus and species of the plant were identified and confirmed. The peel powder was dissolved in 80% ethanol and kept at room temperature for 96 *hr*. Then, the mixture was filtered and concentrated under nearly vacuum pressure at 40*°C* in the rotary evaporator. Four fractions of the crude extract, with different polarities were prepared by in-solution isolation and using the difference in various secondary metabolites’ polarities (Figure 1). The crude extract was dissolved in ethyl alcohol/H_2_O and fractionated by consecutive liquid/liquid partitioning with n-hexane (Merck, Germany), and then with chloroform (Merck, Germany), ethyl acetate (Merck, Germany) and n-butanol (Merck, Germany) with increasing order of polarity 13.

### Determination of total phenolic content

The total phenolic content of pomegranate peel extract and its fractions was determined using Folin-Ciocalteu method 14. Gallic acid was used as a standard reference for plotting calibration curve. Briefly, the dried extract/fractions or gallic acid were dissolved in 80% methanol for preparing 1 *mg/ml* of solutions. 0.2 *ml* of the diluted extract/fractions or standard solution of gallic acid (250, 125, 62.5, 31.2, and 15.6 *μg/ml*) were added to 1 *ml* of 10% (*v/v*) Folin-Ciocalteu reagent and kept at room temperature for 3–8 *min*. Next, 0.8 *ml* of 7.5% (*w/v*) sodium carbonate solution was added to the mixture. After the reaction solution was kept in total darkness for 30 *min*, its optical absorbance was measured at 765 *nm* using a UV-Vis spectrophotometer (UNICO 2100: USA). Total phenolic content was calculated using a gallic acid calibration curve. The results were expressed as *mg* gallic acid equivalents per g of dry plant extract/fractions (mg GAE/g).

### Determination of total flavonoid content

The total flavonoid content of pomegranate peel extract and its fractions was measured according to previously described methods 15. Rutin was used as a standard reference for plotting calibration curve. Briefly, 0.2 *ml* of the diluted extract/fractions (1 *mg/ml*) or standard solution of rutin (125, 62.5, 31.2, 15.6, and 7.8 *μg/ml*) were separately mixed with 0.2 *ml* of 2% (*w/v*) aluminum chloride and 1.2 *ml* of 5% (*w/v*) potassium acetate. After the reaction solution was incubated at Room Temperature (RT) for 40 *min*, its optical absorbance was read at 415 *nm* using a UV-Vis spectrophotometer (UNICO 2100: USA). The results were expressed as *mg* of rutin equivalents per *g* of dry plant matter (*mg* RUT/*g*) in comparison with the standard curve, which was developed under the same conditions.

### Cell culture and influenza virus propagation

Madin-Darby Canine Kidney (MDCK) cell line and influenza virus A/Puerto Rico/8/34 (H1N1; PR8) were obtained from Influenza Unit, Pasteur Institute of Iran. MDCK cells were grown in Dulbecco’s Modified Eagle’s Medium (DMEM) (Gibco, USA), supplemented with 10% Fetal Bovine Serum (FBS) (Gibco, USA) and 1% penicillin streptomycin (Gibco, USA) at 37*°C* in a 5% CO_2_ atmosphere and humidified incubator.

### Cytotoxicity assay

The effect of pomegranate peel extract and its fractions on the viability of MDCK cells was determined using 3-(4,5-dimethylthiazol-2-yl)-2,5-diphenyltetra zoliumbromide (MTT; Sigma, USA) assay, by a previously described method 16 with some modifications. Briefly, when the cell monolayer was confluent, the cells were incubated with 200 *μl/well* of various concentrations of the extract/fractions (200, 100, 50, 25, 12.5, 6.25 and 3.1 *μg/ml*) in 96-well plates for 48 *hr*. Afterwards, cell monolayers were incubated with 50 *μl* of 1 *mg/ml* MTT in Phosphate-Buffered Saline (PBS) at 37*^o^C* for 4 *hr*, and then treated with 100 *μl* of acidic isopropanol (0.05 *N* HCl in absolute isopropanol). After the plates were shaken for 15 *min*, the absorbance was read using a reference filter at 640 *nm* using microplate reader (StataFax2100, USA).

### Cytopathic effect (CPE) reduction assay

When the cell monolayer was confluent in 96-well plates, the cell culture medium was aspirated and washed with PBS and infected with 100TCID_50_ of *Influenza A* (H1N1) virus for 1 *hr*, and then the virus was removed and the cells were treated with serial two fold dilutions of nontoxic concentration of the extract/fractions (200 *μl/well*) in serum-free DMEM containing 2 *μg/ml* of TPCK-trypsin and 0.3% BSA. 48 *hr* post infection, cell viability was also determined using previously described MTT assay 16. Various concentrations of oseltamivir (10, 5, 2.5, 1.25, 0.62 and 0.31 *μmol*) (Sigma, USA) were used as positive controls. The procedure was carried out in triplicate. The 50% cytotoxic concentration (CC_50_) and 50% inhibitory concentration (IC_50_) were calculated using GraphPad Prism 6 (Graph-Pad Software, La Jolla, CA). Selectivity index (SI) was calculated as ratio of CC_50_ to IC_50_.

### Hemagglutination assay

MDCK cells in 24-well plates were infected with PR8 virus at 100 TCID_50_, incubated with virus for 1 *hr* at 37°*C* and cultured in DMEM and TPCK trypsin (0.5 *μg/ml*; Sigma, USA) either with or without extract/fractions treatment. The cell culture supernatants were harvested 24 and 48 *hr* post infection. Fifty *μl* of the two fold serial dilutions of the cell culture supernatants were mixed with the same volume of 0.5% chicken Red Blood Cells (RBCs) in U-bottomed 96-well plate for 45 *min* at room temperature. The HA was performed by measuring the dilution factor of the samples required for complete HA-mediated chicken RBC agglutination 17.

### TCID_50_ virus titration

Confluent MDCK cells monolayer in 24-well plates were infected with PR8 virus (100 TCID_50_) in the presence of the extract/fractions or control compounds for 24 *hr* at 37*oC*. Standard 50% Tissue Culture Infectious Doses (TCID_50_) were used for virus titration in culture supernatants 18. Briefly, when 90%-confluent MDCK cells were prepared in 96 well plates, the cell culture medium was aspirated and washed with PBS twice, and then 100 *μl* of a series of 10-fold dilutions was added into the wells and left to incubate for 2 days. After the incubation, virus replication was detected by HA 18–20. TCID_50_ calculated based on the Reed and Muench method was expressed as log10 21.

### Statistical analysis

The data were analyzed using Kruskal-Wallis test to compare differences between the groups. The IC_50_ and CC_50_ values were calculated by regression analysis using GraphPad Prism 6 (GraphPad Software, La Jolla, CA).

## Results

### Total phenolic and flavonoid content

The results on total phenolic and total flavonoid content in the extract and fractions are presented in table 1. The results showed that pomegranate peel extract was the richest source of phenolic (233 *mg GAE/g*) and flavonoid (60.6 *mg RUT/g*) content. The highest phenolic (692 *mg GAE/g*) and flavonoid (93.7 *mg RUT/g*) content was obtained for ethyl acetate fraction (Table 1).

**Table 1.** Total phenolic and flavonoid values of pomegranate peel crude extract and its fractions

**Sample**	Total phenolics (mg GAE/g)^*^	Flavonoid content (mg RUT/g)^**^
**Crude extract**	233±2.4	60.6.1±1.4
**n-Hexan fraction**	22.1±1.5	11.5±2.75
**Choloroform fraction**	221.9±3.5	83.2±1.9
**Ethyl acetate fraction**	476±4.2	60.6±2.7
**n-Butanol fraction**	199±1.8	77.1±2.5
**p-value^#^**	< 0.05	< 0.05

* *mg* gallic acid equivalent/g of extract/fractions,

** *mg* rutin equivalent/g of extract/fractions;

# according to Kruskal-Wallis; all results are presented as mean (± standard error of the three measurements).

### Cytotoxicity and anti-influenza A virus activity

**Figure 2.**
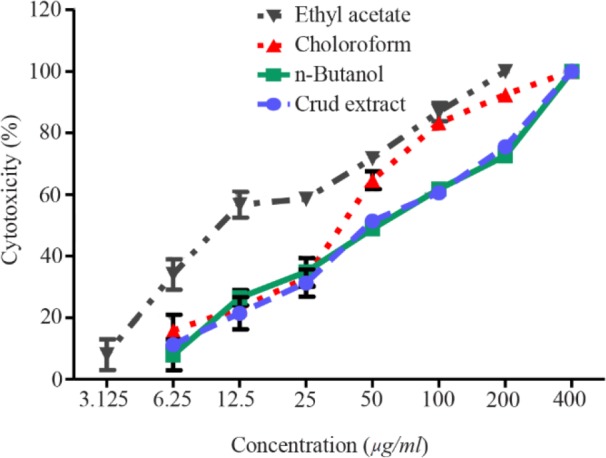
Cytotoxicity of pomegranate peel extract and its fractions on MDCK cells. Confluent MDCK cells were exposed to different concentrations of crude extract and its fractions for 48 *hr*. Cytotoxi-city was measured in MTT assay; experiments were carried out in triplicate.

Based on the CPE reduction assay results and probit analysis, the CC_50_ of crude extract and n-butanol and ethyl acetate fractions was 55.66, 55.61, and 29.7 *μg/ml*, respectively (Table 2). The analysis showed that there was a direct, significant relationship between the concentration of the extract/fractions and cell death (p< 0.05, Figure 2). The antiviral activities of the extract and the four fractions against the influenza A/PR/8/34 virus were investigated 48 *hr* after treatment using an MTT-based CPE reduction assay. Results indicated that the crude extract and n-butanol and ethyl acetate fractions produced antiviral effect against influenza virus with the SIs of 8.63, 9.16 and 5.3, respectively (Table 2).

**Table 2.** Cell cytotoxicity and anti-influenza virus activity of pomegranate peel extract and fractions

**Extract/fractions**	**CC_50_^a^*μg/ml* (CI95%)**	**IC_50_^b^*μg/ml* (CI95%)**	**SI^c^**
**Crude extract**	55.6 (48.4–64)	6.4 (4.5–9.2)	8.63
**n-hexane fraction**	238.2 (142.4–398.4)	>238.2	-
**Chloroform fraction**	34.1 (30.1–38.6)	>34.1	-
**Ethyl acetate fraction**	29.7(24.9–35.3)	5.6 (3.9–7.9)	5.3
**n-butanol fraction**	55.61 (47.1–65.6)	6.1 (4.5–8.13)	9.16
Oseltamivir (*μmol*)^*^	539.4 (378.9–768.5)	0.87 (0.55–1.4)	617.8

a CC_50_: 50% cytotoxic concentration (MDCK cell);

b IC_50_: 50% inhibitory concentration (PR8 influenza virus);

c SI: Selectivity index, *i.e*., the ratio of CC_50_ to IC_50_; CI95%: 95% confidence interval;

* oseltamivir used as positive control.

### Inhibition of influenza virus replication

Based on the CPE reduction assay results (Table 2), the crude extract and the n-butanol and ethyl acetate fractions underwent additional antiviral assays. The HA titers of the extract and the fractions on influenza virus were assessed by hemagglutination endpoint test. According to the results, the viral titer decreased dose dependently after treatment with the crude extract and the n-butanol and ethyl acetate fractions (Table 3).

**Table 3.** Hemagglutination titers of PR8-infected MDCK cell supernatants in the presence of the pomegranate peel extract and its more effective fractions

**Extract/fractions**	**Concentration (*μg/ml*)**	**Log2 HA titer/50 *μl* supernatant**			

		**24 *hr*^a^**	**48 *hr*^a^**
**Crude extract**			
	50	0	0
	25	0.67±1.15	4±3.46
	12.5	1.67±1.15	6.33±0.58
	6.25	2.67±1.15	6.67±1.15
	virus control	5±1.4	7.33±.58
**n-butanol fraction**			
	50	0	0
	25	0	2.5±0.71
	12.5	1.5±0.71	6
	6.25	3.5±0.71	6.5±0.71
	virus control	6	7.5±0.71
**Ethyl acetate fraction**			
	25	0	1±1.73
	12.5	0.33±0.58	4±1.43
	6.25	1.67±1.53	6.33±0.58
	3.12	2.67±0.58	6.67±1.15
	virus control	4±1.73	7.33±0.58
**Oseltamivir (*μmol*)^b^**			
	5	0	0
	2.5	0	0.5±0.71
	1.25	0	4
	virus control	5±1.4	8

a: Hours post-infection;

b: Oseltamivir used as positive control.

**Figure 3.**
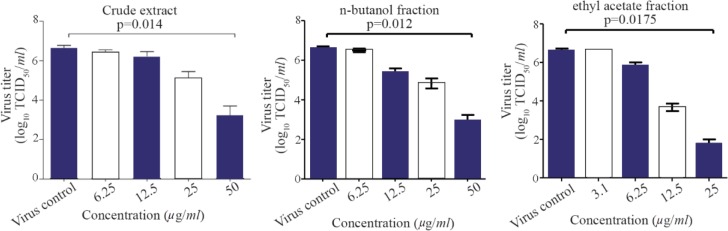
Reduction of influenza viral titers in the culture supernatants by the pomegranate peel extract and its more effective fractions. PR8-infected MDCK cells were incubated with different concentrations of the extract/fractions for 24 *hr* and the supernatants were used for TCID_50_ titration. The data are the mean values of three independent experiments (mean±SEM). p-values were calculated against virus control (untreated sample) using Kruskal-Wallis test.

To investigate whether pomegranate peel extract and n-butanol fraction and ethyl acetate fraction could have inhibitory effect on infectious virus yield, the virus was titrated by TCID_50_ method. Consistent with the results of the HA, the production of virus was significantly reduced upon treatment with pomegranate peel extract and n-butanol and ethyl acetate fractions in a dose-dependent manner (p<0.05; Figure 3).

## Discussion

Pomegranate is a highly active and important medicinal plant in folk medicine and its antibacterial, antiparasitic, apoptotic, antifungal, antiproliferative, and anti-viral activities have recently been studied 22–24.

Although few studies reported the inhibitory effects of pomegranate fruit against herpes virus, influenza virus, poxviruses, and human immunodeficiency virus 24,25, this is the first report on the antiviral activity of corresponding fractions of pomegranate peel. Our aim, therefore, was to study the anti-influenza activity of pomegranate peel extract and its fractions in the MDCK cell line.

In the present study, the crude extract inhibited influenza A PR8 virus replication in the MDCK cell line [IC_50_: approximately 6.45 (4.5–9.23)]. According to the results of antiviral assays to measure the titers of HA or infectious viral particles in the culture supernatants, it was observed that pomegranate peel could suppress the amplification of the infectious influenza viruses. Because the IC_50_ of an herbal extract for infectious diseases is conventionally less than 100 *μg/ml* 26 and SI over 4 27, pomegranate peel extract with IC_50_ of 6.45 and SI of 8.63 can be considered a potent agent to fight influenza virus.

Our results showed that the crude extract and n-butanol and the ethyl acetate fractions exerted more potent antiviral effects than other fractions. Other studies have also shown that the antiviral property of pomegranate extract may be due to hydrolysable tannins and polyphenols, especially punicalagin and gallagic acid, which have also been found in this extract 23. A study indicated that out of the four flavonoids of pomegranate, *i.e*. ellagic acid, caffeic acid, luteolin, and punicalagin, only punicalagin had inhibitory effect against influenza virus 25. Pomegranate peel’s polyphenol compounds such as punicalagin, ellagic acid, and hydroxy-benzoic acid that are extracted from both n-butanol and ethyl acetate fractions are probably associated with antiviral activity of pomegranate peel.

## Conclusion

Based on our results, both n-butanol and ethyl acetate fractions of pomegranate peel, with high inhibitory effect against influenza virus replication, could be a new promising anti-influenza agent. More understanding of the mechanism of action and the natural components of these fractions seems necessary. The results of this study also showed the presence of high amounts of polyphenols in the fractions. As a result, the antiviral activity of the plant could be partly attributed to its polyphenol content.
